# Pandemieassoziierte Konsequenzen und Unterstützungsbedarf: Mixed-methods-Längsschnittanalyse über 2 Jahre der COVID-19-Pandemie in Deutschland

**DOI:** 10.1007/s00278-022-00631-9

**Published:** 2022-12-29

**Authors:** Antonia Bendau, Jens Plag, Luise Schulz, Moritz Bruno Petzold, Andreas Ströhle

**Affiliations:** 1 Charité – Universitätsmedizin Berlin, Corporate member of Freie Universität Berlin and Humboldt Universität zu Berlin, Klinik für Psychiatrie und Psychotherapie, CCM, Charitéplatz 1, 10117 Berlin, Deutschland; 2 HMU Health and Medical University Potsdam, Potsdam, Deutschland; 3 Oberberg Fachklinik Potsdam, Potsdam, Deutschland; 4 Fachbereich Erziehungswissenschaft und Psychologie, Freie Universität Berlin, Berlin, Deutschland

**Keywords:** Emotionaler Stress, Seelische Belastung, Psychische Gesundheit, Angst, Corona, Emotional stress, Mental strain, Mental health, Anxiety, Corona

## Abstract

**Hintergrund:**

Die COVID-19-Pandemie geht potenziell mit zahlreichen Veränderungen im Leben der Allgemeinbevölkerung einher. Dennoch gibt es bisher kaum Befunde dazu, welche Auswirkungen als besonders gravierend und negativ erlebt wurden, wie sich diese Auswirkungen und ihre Bewertungen im Verlauf der Pandemie änderten, und welche Unterstützungswünsche in diesem Kontext entstanden.

**Methode:**

Längsschnittliche Daten einer Onlinestudie mit 10 Messzeitpunkten über die ersten 2 Jahre der Pandemie (März 2020 bis April 2022) wurden in einer Mixed-methods-Analyse betrachtet. Die Stichprobe aus 8337 Erwachsenen der Allgemeinbevölkerung in Deutschland beantwortete qualitative Freitextfragen zu den gravierendsten Konsequenzen der Pandemie auf ihr Leben sowie Unterstützungswünschen. Quantitativ wurden zudem die Bewertung der Konsequenzen im Pandemieverlauf und ihre Assoziationen mit psychischer Belastung betrachtet.

**Ergebnisse:**

Die erlebten Konsequenzen und insbesondere ihre Bewertung veränderten sich im Pandemieverlauf. Sozial-gesellschaftliche und das allgemeine Leben betreffende Konsequenzen wurden im Schnitt besonders gravierend und negativ erlebt. Negativer erlebte Konsequenzen waren quer- und teilweise auch längsschnittlich mit stärkeren Angst- und depressiven Symptomen assoziiert. Psychotherapeutische sowie evaluativ-kommunikative Unterstützung wurde im Pandemiekontext besonders häufig erbeten.

**Schlussfolgerungen:**

Subjektiv negativ erlebte Konsequenzen sollten möglichst durch adäquate Maßnahmen abgemildert werden. Die dynamischen Veränderungen der Konsequenzen und damit auch des Unterstützungsbedarfs im Pandemieverlauf sollten berücksichtigt werden. Die Unterstützungsmöglichkeiten reichen von sehr niedrigschwelligen Angeboten (z. B. Tipps online) bis hin zu einer Psychotherapie.

## Abstract (en)

**Background:**

The coronavirus disease 2019 (COVID-19) pandemic is potentially associated with numerous changes in the life of the general population; nevertheless, to date there is hardly any evidence on which effects are experienced as particularly severe and negative, how these effects and their evaluations changed over the course of the pandemic and which wishes for support arose in this context.

**Method:**

Longitudinal data from an online study with 10 assessments over the first 2 years of the pandemic (March 2020–April 2022) were analyzed in a mixed methods approach. The sample of 8337 adults from the general population in Germany answered qualitative free text questions about the most severe consequences of the pandemic on their lives and need/wishes for support. In addition, the evaluation of the consequences over the course of the pandemic and their associations with psychological distress were quantitatively examined.

**Results:**

The consequences experienced and especially their evaluation changed over the course of the pandemic. On average, consequences targeting social and life in general aspects were experienced as particularly severe and negative. Negatively experienced consequences were cross-sectionally and partly also longitudinally associated with stronger anxiety and depressive symptoms. Psychotherapeutic and evaluative communicative support was particularly frequently requested in the context of the pandemic.

**Conclusion:**

Subjectively negatively experienced consequences of the pandemic should if possible be mitigated by adequate measures. The dynamic changes of the consequences and thus also the need for support over the course of the pandemic should be taken into account. Possible support options range from very low-threshold offers (e.g., tips online) up to psychotherapy.

Eine detaillierte Analyse dessen, welche Konsequenzen der COVID-19-Pandemie besonders gravierend erlebt werden, wie diese bewertet werden, und wie sich dies im Pandemieverlauf verändert, fehlte bislang. Auch über konkrete Unterstützungswünsche im Kontext der Pandemie ist noch relativ wenig bekannt. Eine unvoreingenommene und gesamtheitliche Erfassung von Unterstützungswünschen sowie erlebten Pandemiekonsequenzen erscheint wichtig, um die subjektiv erlebten Auswirkungen in ihrer Vielschichtigkeit abzubilden und daraus Implikationen für weitere Forschung sowie präventive und therapeutische Maßnahmen abzuleiten.

## Hintergrund und Fragestellung

*„*Das Coronavirus verändert zurzeit das Leben in unserem Land dramatisch“*, *postulierte Angela Merkel zu Beginn der COVID-19-Pandemie in ihrer Ansprache an die Bevölkerung in Deutschland (Bundesregierung 2020). Während sich in den zweieinhalb Jahren seitdem Studien mehrten, die sich verschiedenen pandemiebedingten Belastungen widmen, fehlt jedoch eine detaillierte Analyse dessen, welche Konsequenzen der Pandemie besonders gravierend erlebt, wie diese bewertet werden, und wie sich dies im Pandemieverlauf verändert. Auch über konkrete Unterstützungswünsche im Kontext der Pandemie ist bisher noch relativ wenig bekannt.

Das dynamische Infektionsgeschehen sowie die restriktiven Maßnahmen zur Eindämmung der Pandemie können sich auf zahlreiche Bereiche des Lebens auswirken (Cénat et al. 2021; Schafer et al. 2022; Bourmistrova et al. 2022). Insbesondere zu Beginn der Pandemie wurden derartige Auswirkungen in vielfältigen Untersuchungen rund um den Globus beobachtet. Besonders deutliche Pandemieauswirkungen wurden beispielsweise in Bezug auf das Berufsleben (Homeoffice, Kurzarbeit, Einkommensreduktion) sowie das soziale Miteinander (Kontaktbeschränkungen, Quarantäne, Isolation) ersichtlich (Entringer und Gosling 2022; Kühne et al. 2020; Bueno-Notivol et al. 2021; Cornesse et al. 2022). Parallel stellten zahlreiche Studien im Kontext der Pandemie einen Anstieg in Symptomen von Angst, Stress, Depression, Schlafstörungen und weiteren Belastungsreaktionen fest – häufig besonders ausgeprägt zu Pandemiebeginn (Cénat et al. 2021; Schafer et al. 2022; de Sousa et al. 2021; Bendau et al. 2021a, 2020). Der Schweregrad dieser psychischen Symptome war wiederum assoziiert mit pandemiebedingten Faktoren, wie beispielsweise Quarantäne (Henssler et al. 2021), einer Infektion mit dem „severe acute respiratory syndrome coronavirus 2“ (SARS-CoV-2) bei sich oder Angehörigen (Shevlin et al. 2020) und beruflichen Veränderungen sowie finanziellen Schwierigkeiten (Leung et al. 2022).

Meist wurden diese Auswirkungen und Assoziationen mit psychischer Belastung bisher nur in Querschnittsanalysen betrachtet (Cénat et al. 2021; Henssler et al. 2021); deren Aussagekraft ist gerade in Anbetracht der hohen Dynamik der Pandemie mit stark fluktuierenden Infektionsraten und Restriktionen eingeschränkt. Daher erscheinen längsschnittliche Studien dringend notwendig. Die vorhandenen Studien fokussieren zudem mehrheitlich ausschließlich auf negative Konsequenzen, während auch positive Auswirkungen der Pandemie durchaus möglich sind (Bendau et al. 2021a). Des Weiteren begrenzten sich bisherige Studien im Rahmen quantitativer Designs mit vorgegebenen Items zu bestimmten Konsequenzen überwiegend auf einzelne vorab definierte Lebensbereiche, wodurch eine unvoreingenommene und gesamtheitliche Erfassung erlebter Pandemiekonsequenzen bisher vernachlässigt wurde. Genau dies scheint aber wichtig, um die subjektiv erlebten Auswirkungen in ihrer Vielschichtigkeit korrekt abzubilden und daraus Implikationen für weitere Forschung sowie präventive und therapeutische Maßnahmen abzuleiten.

Aus diesem Bestreben ergab sich die Zielstellung der vorliegenden Arbeit in einem Mixed-methods-Ansatz, qualitativ die gravierendsten subjektiv wahrgenommenen Auswirkungen der Pandemie zu analysieren und außerdem quantitativ die Valenz der subjektiven Bewertung der bisherigen Konsequenzen auf verschiedenen Dimensionen im Pandemieverlauf sowie deren Assoziationen mit psychischer Belastung zu untersuchen.

Des Weiteren widmet sich diese Arbeit der Fragestellung, ob und welche Form der psychiatrisch-psychotherapeutischen oder anderweitigen Unterstützung sich belastete Menschen im Kontext der Pandemie wünschen. Denn auch hierzu gibt es bisher nur sehr begrenzte Evidenz, die sich auf bestimmte Zielgruppen, wie z. B. Pflegepersonal, konzentriert (Wolf-Ostermann et al. 2020).

## Methoden

**Figure 1:**
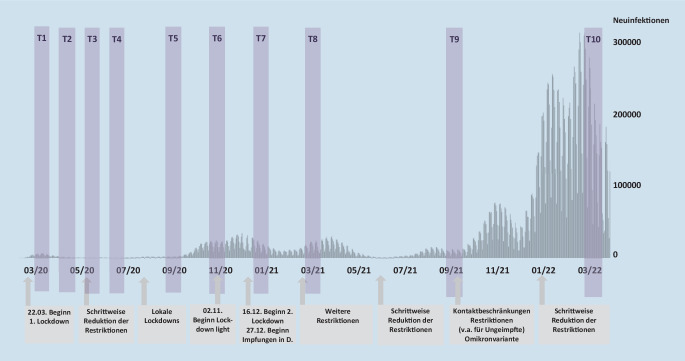

Ein Längsschnittprojekt mit 10 Messzeitpunkten über die ersten 2 Jahre der Pandemie (März/April 2020 bis März/April 2022) lieferte die Datenbasis für die nachfolgenden Analysen (Bendau et al. 2020, 2021b). Eine Gelegenheitsstichprobe aus insgesamt 8337 Erwachsenen der Allgemeinbevölkerung in Deutschland nahm konsekutiv an bis zu 10 Online-Erhebungswellen über die Plattform SoSci-Survey teil (für einen Überblick über die Messzeitpunkte und die Pandemiesituation: Abb. 1). Das Projekt wurde im Vorfeld von der Ethikkommission der Charité – Universitätsmedizin Berlin (EA1/071/20) genehmigt und auf clinicaltrials.gov registriert (NCT04331106).

Die nonprobabilistische Rekrutierung der Proband:innen erfolgte über Nachrichtenportale, Social-Media-Kanäle und die Homepage der Charité – Universitätsmedizin Berlin (Bendau et al. 2020, 2021b; Petzold et al. 2020). Volljährigkeit, ein aktueller Wohnsitz in Deutschland und ausreichende Deutschkenntnisse stellten die Einschlusskriterien dar; die Teilnahme wurde nicht vergütet. Alle Proband:innen erklärten vor der Studienteilnahme ihr informiertes Einverständnis; die Datenerhebung sowie das längsschnittliche Zusammenfügen der einzelnen Messzeitpunkte erfolgten vollständig anonym.

Auf quantitativer Ebene wurden die in den vergangenen 3 Wochen erlebten Konsequenzen der Pandemie auf das eigene Leben zu jedem Messzeitpunkt mit 6 fünfstufig Likert-skalierten Items (von „sehr negativ“ [−2] bis „sehr positiv“ [+2]) subjektiv beurteilt. Hierbei wurde differenziert in Auswirkungen auf das eigene Leben allgemein, gesundheitliche Konsequenzen für die befragte Person selbst, gesundheitliche Konsequenzen für Angehörige, soziale Auswirkungen auf das eigene Leben sowie wirtschaftliche Auswirkungen auf die Person selbst. Zudem wurde die Valenz der bisher eingetretenen Konsequenzen im Vergleich zu den eigenen Erwartungen/Befürchtungen erfasst. Auf qualitativer Ebene wurde des Weiteren von T2 an die bisher gravierendste Auswirkung der Pandemie auf das eigene Leben in einem Freitextfeld erfasst.

Mit einem dichotomen Einzelitem wurde erhoben, ob psychiatrisch-psychotherapeutische oder anderweitige Unterstützung im Umgang mit pandemiebedingten Belastungen gewünscht sei (ja/nein). In einem qualitativen Freitextitem wurden diese Unterstützungswünsche/-bedürfnisse konkretisierend erfragt.

Mittels der 4‑Item-Kurzversion des *Patient Health Questionnaire *(PHQ‑4; Löwe et al. 2010) wurden Kernsymptome generalisierter/unspezifischer Angst (Subskala GAD-2) sowie depressive Symptome (Subskala PHQ-2) erfasst.

Die qualitative Analyse der Freitextantworten auf die Frage nach der gravierendsten Auswirkung erfolgte orientiert an der *qualitativen Inhaltsanalyse* (QIA, Kuckartz 2018; Mayring 2015) mithilfe der etablierten Kodiersoftware MaxQDA 2020 (VERBI Software, Consult, Sozialforschung GmbH, Berlin, Deutschland). Im ersten Materialdurchgang wurden zunächst am Datenmaterial induktiv Kategorien gebildet, um eine unvoreingenommene Abbildung des Materials zu ermöglichen (Willems et al. 2020; Kuckartz 2018; Mayring 2015). Im Kategorienbildungsvorgang wurde das Textmaterial gemäß Mayring (2015) Zeile für Zeile durchgearbeitet, und die Antworten wurden per Kodierfunktion jeweils der bestpassenden Kategorie zugeordnet. Das Kategoriensystem galt als fertiggestellt, sobald kaum noch neue Kategorien hinzukamen (nach Durcharbeitung etwa 30 % des Materials; Kuckartz 2018) und wurde anschließend gemäß Mayring (2015) revidiert. In einem zweiten Durchlauf wurde das gesamte Textmaterial mit den gebildeten Kategorien kodiert (Mayring 2015). Die zentralen Gütekriterien Intra- und Interkoder-Übereinstimmung wurden durch einen weiteren unvoreingenommenen Durchlauf sowie den Abgleich mit einer unabhängigen Zweitkodiererin überprüft (Mayring und Fenzl 2019). Im nächsten Schritt wurden die 20 gebildeten Kategorien deduktiv auf Grundlage des vorhandenen Kategoriensystems zu 5 Hauptkategorien zusammengefasst (Mayring 2015; Kuckartz 2018). Analog war das Vorgehen bezüglich des Unterstützungswunsches, wobei die 10 finalen Kategorien nicht in übergeordneten Hauptkategorien deduktiv gebündelt wurden.

Alle quantitativen Analysen wurden mit dem Statistikprogramm IBM SPSS Statistics Version 27 (IBM Corp., Armonk, NY, USA) ausgeführt. Neben deskriptiven Analysen wurden für 3 ausgewählte Messzeitpunkte (T1 [Baseline]: 27.03.2020–06.04.2020; T2 [Ein-Monats-Follow-up]: 24.04.2020–04.05.2020; T3: [Ein-Jahres-Follow-up]: 26.03.2021–05.04.2021) inferenzstatistisch querschnittliche sowie längsschnittlich-prospektive (für Baseline-Korrelationen kontrollierte) Zusammenhänge der Valenz der erlebten Konsequenzen mit Angst- und depressiven Symptomen berechnet. Das Signifikanzniveau (zweiseitig) wurde auf 0,05 festgesetzt.

## Ergebnisse

### Stichprobe

Von den insgesamt 8337 Teilnehmenden identifizierten sich 71,7 % (*n* = 5980) als weiblich, 27,6 % (*n* = 2297) als männlich und 0,7 % (*n* = 60) als divers. Das Durchschnittsalter bei der Erstteilnahme an der Studie betrug 37,54 Jahre (SD ± 12,04 Jahre; Range 18 bis 99 Jahre). Bezüglich des Bildungsniveaus gaben 53,3 % einen Universitätsabschluss, 30,1 % eine (Fach‑)Hochschulreife und 16,6 % einen Realschulabschluss oder niedriger an. Ein Prozentsatz von 9,5 % litt unter (chronischen) körperlichen Erkrankungen (z. B. Krebs, Herz-Kreislauf-Erkrankungen, Stoffwechselerkrankungen). Die Anteile der Individuen, die mit SARS-CoV‑2 infizierte Personen kannten (26,8 % zu T1; 98,7 % zu T10), bereits selbst infiziert waren (0,9 % zu T1; 28,4 % zu T10) sowie mindestens eine COVID-19-Impfung erhalten hatten (T8: 24,3 % zu T10: 96,3 %), stiegen kontinuierlich über die 10 Messzeitpunkte an.

### Konsequenzen der Pandemie auf das eigene Leben

**Figure 2:**
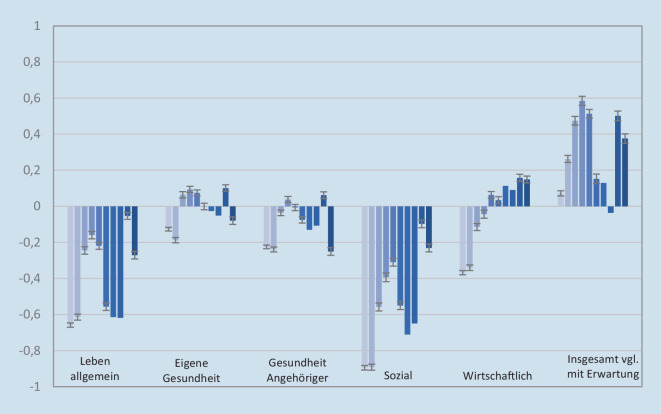

In Abb. 2 wird illustriert, wie sich die Bewertung verschiedener individuell erlebter Konsequenzen der Pandemie im Verlauf veränderte. Auf Mittelwertsebene fielen insbesondere die Bewertung der Konsequenzen auf das Leben i. Allg. sowie der sozialen Folgen negativ aus – gerade zu Beginn sowie während Phasen mit hohen Infektionszahlen und strengen restriktiven Maßnahmen. Gesundheitsbezogene Konsequenzen wurden dagegen im Schnitt relativ neutral beurteilt und wirtschaftliche Konsequenzen zunehmend positiver. Interessanterweise wurde im Mittel angegeben, dass die Konsequenzen insgesamt etwas positiver ausgefallen seien als erwartet. In allen Items wurde ein Range von sehr negativen bis hin zu sehr positiven Auswirkungen ersichtlich.

**Figure 3:**
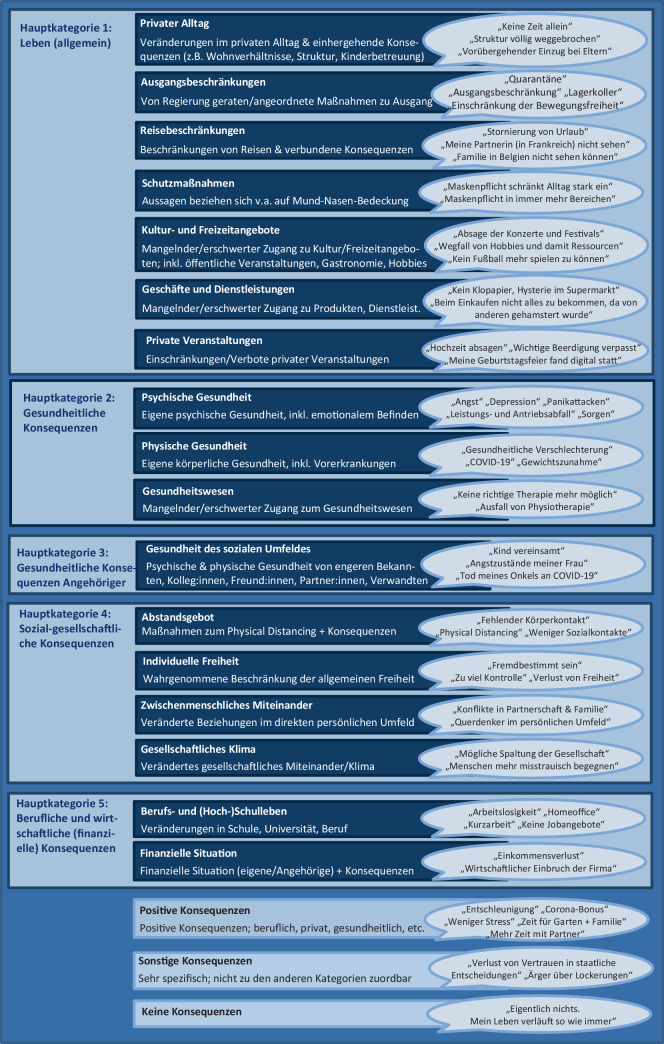

**Figure 4:**
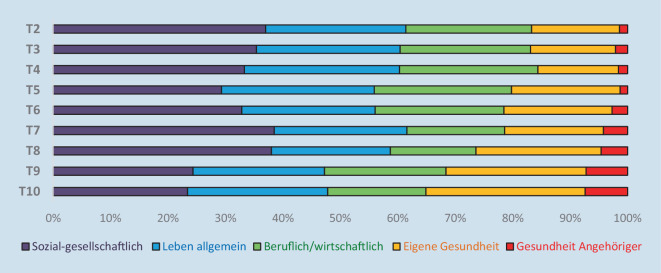

Aus der qualitativen Analyse ergaben sich insgesamt 20 Kategorien, die in 5 Hauptkategorien zusammengefasst wurden (Abb. 3). Am häufigsten wurden zu allen Messzeitpunkten Konsequenzen, die der sozial-gesellschaftlichen Hauptkategorie zugeordnet werden können, als am gravierendsten genannt (Abb. 4). Die am häufigsten genannte Subkategorie über die Messzeitpunkte hinweg stellte das *Abstandsgebot (Physical Distancing; soziale Isolation)* dar (15,2–31,5 %), gefolgt von *Berufs- und (Hoch)Schulleben* (13,5–19,2 %) und *psychischer Gesundheit* (9,0–14,1 %). Auch *positive Konsequenzen* wurden teilweise als gravierendste Auswirkungen genannt (2,1–4,8 %).

Die negativere Bewertung der erlebten Konsequenzen zu T1 korrelierte querschnittlich mit erhöhten Angst- sowie depressiven Symptomen (Tab. 1). Auch längsschnittlich (bei Herausrechnung der T1-Querschnittkorrelation) sagten negativere Konsequenzen auf das Leben allgemein sowie auf die Gesundheit Angehöriger höhere psychische Belastung einen Monat später (T2) vorher. Eine negativere Bewertung der erlebten allgemeinen, sozial-gesellschaftlichen sowie wirtschaftlichen Konsequenzen korrelierte des Weiteren mit vermehrter psychischer Belastung ein Jahr später (T8). Die (inkrementelle) Varianzaufklärung der psychischen Belastung durch die Konsequenzen fiel in den statistisch signifikanten querschnittlichen (Range R^2^: 0,6–11,8 %) und insbesondere den längsschnittlichen Zusammenhängen (Range R^2^: 0,3–1 %) allerdings relativ gering aus; die Effektstärke dieser Zusammenhänge ist also als klein zu bewerten.

**Table 1:** 

Valenz erlebter Konsequenzen T1		Querschnitt	Längsschnittanalyse	
		T1 2020 27.03.2020–06.04.2020 (*n* = 5114)	T2 2020 24.04.2020–04.05.2020 (*n* = 1326)	T8 2021 26.03.2021–05.04.2021 (*n* = 859)
Leben, allgemein	Angst (GAD-2)	**β** **=** **−0,321 (*p*** **<** **0,001) R**^**2**^ **=** **0,103**	**β** **=** **−0,097 (*p*** ***=*** **0,010)** **+R**^**2**^ **=** **0,004**	β = −0,005 (*p* = 0,905)
	Depression (PHQ-2)	**β** **=** **−0,343 (*p*** **<** **0,001) R**^**2**^ **=** **0,118**	**β** **=** **−0,071 (*p*** ***=*** **0,050)** **+R**^**2**^ **=** **0,003**	**β** **=** **−0,082 (*p*** **=** **0,037)** **+R**^**2**^ **=** **0,005**
Eigene Gesundheit	Angst (GAD-2)	**β** **=** **−0,243 (*p*** **<** **0,001) R**^**2**^ **=** **0,059**	β = −0,063 (*p* = 0,107)	β = −0,049 (*p* = 0,252)
	Depression (PHQ-2)	**β** **=** **−0,227 (*p*** **<** **0,001) R**^**2**^ **=** **0,052**	β = −0,001 (*p* = 0,976)	β = 0,015 (*p* = 0,713)
Gesundheit, Angehöriger	Angst (GAD-2)	**β** **=** **−0,078 (*p*** **<** **0,001) R**^**2**^ **=** **0,006**	**β** **=** **−0,079 (*p*** ***=*** **0,044)** **+R**^**2**^ **=** **0,003**	β = −0,024 (*p* = 0,581)
	Depression (PHQ-2)	**β** **=** **−0,076 (*p*** **<** **0,001) R**^**2**^ **=** **0,006**	β = 0,008 (*p* = 0,835)	β = −0,016 (*p* = 0,689)
Sozial-gesellschaftlich	Angst (GAD-2)	**β** **=** **−0,178 (*p*** **<** **0,001) R**^**2**^ **=** **0,032**	β = −0,071 (*p* = 0,064)	β = −0,072 (*p* = 0,089)
	Depression (PHQ-2)	**β** **=** **−0,249 (*p*** **<** **0,001) R**^**2**^ **=** **0,062**	β = −0,035 (*p* = 0,337)	**β** **=** **−0,121 (*p*** ***=*** **0,002)** **+R**^**2**^ **=** **0,010**
Wirtschaftlich	Angst (GAD-2)	**β** **=** **−0,094 (*p*** **<** **0,001) R**^**2**^ **=** **0,009**	β = −0,002 (*p* = 0,956)	**β** **=** **−0,115 (*p*** ***=*** **0,008)** **+R**^**2**^ **=** **0,009**
	Depression (PHQ-2)	**β** **=** **−0,118 (*p*** **<** **0,001) R**^**2**^ **=** **0,014**	β = −0,007 (*p* = 0,848)	β = −0,046 (*p* = 0,262)

*β* standardisierte Regressionskoeffizienten der linearen Regression der psychischen Belastung (Generalized Anxiety Disorder Scale‑2, *GAD‑2*; Patient Health Questionnaire‑2, *PHQ‑2*) auf die erlebten Konsequenzen; signifikante Zusammenhänge fett gedruckt; bei statistischer Signifikanz wurde R^2^ als Information bezüglich der (inkrementellen) Varianzaufklärung ergänzt. Um die Anzahl multipler Tests gering zu halten, wurden exemplarisch nur 3 Messzeitpunkte ausgewählt; T1 für querschnittliche Assoziationen sowie T2 (einen Monat später) und T8 (ein Jahr später) für längsschnittliche Assoziationen bei Kontrolle der Baseline-Querschnittskorrelationen

### Unterstützungswunsch

**Figure 5:**
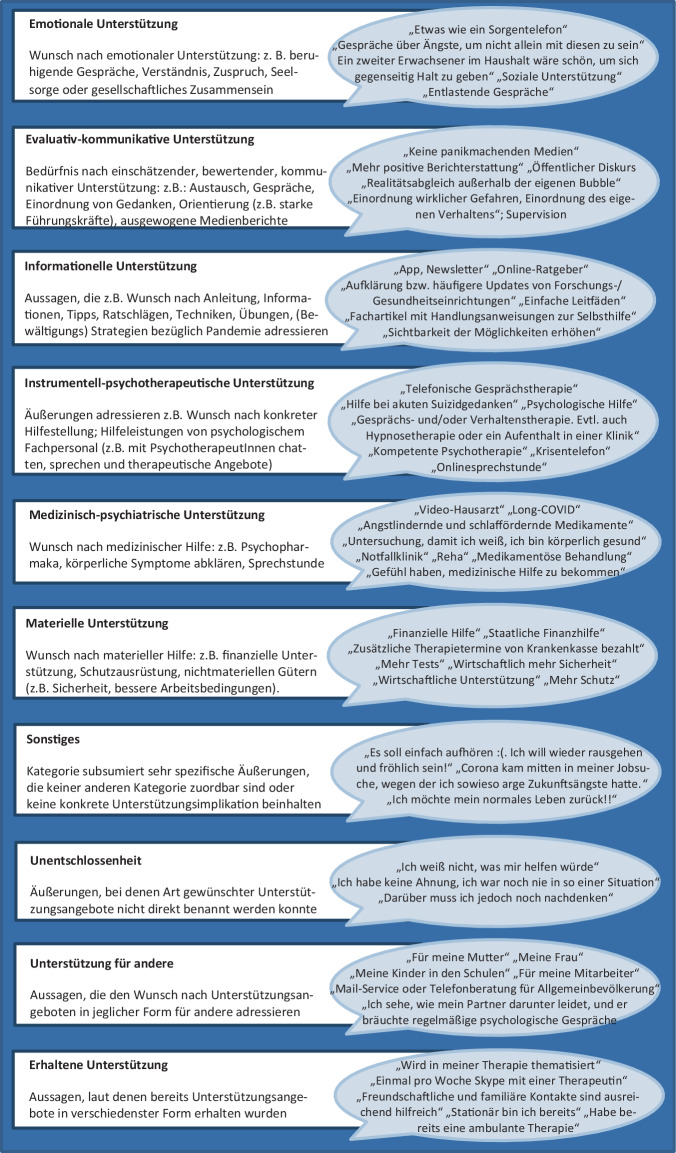

**Figure 6:**
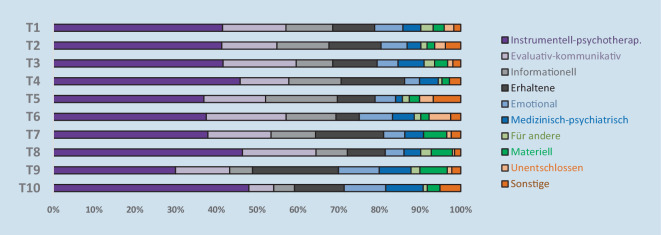

Der Anteil der Teilnehmenden, die sich psychiatrisch-psychotherapeutische oder anderweitige Unterstützung im Umgang mit Ängsten und Belastung in der Pandemie wünschten, schwankte leicht zwischen den Messzeitpunkten (T1: 13,1 %; T2: 11,7 %; T3: 10,8 %; T4: 8,9 %; T5: 10,0 %; T6: 13,8 %; T7: 11,7 %; T8: 15,2 %; T9: 8,2 %; T10: 8,9 %). Im Rahmen der qualitativen Auswertung wurden 10 Kategorien für den Wunsch/Bedarf nach Unterstützung gebildet (Abb. 5). Am häufigsten wurde der Wunsch nach psychotherapeutischer Unterstützung geäußert, gefolgt von evaluativ-kommunikativen und informationellen Aspekten (Abb. 6).

## Diskussion

### Zusammenfassung und Interpretation der Befunde

Die vorliegende Mixed-methods-Studie untersuchte Konsequenzen und Unterstützungswünsche/-bedarf im Kontext der COVID-19-Pandemie in einem Längsschnittprojekt über 2 Jahre hinweg. Insbesondere den sozial-gesellschaftlichen Bereich sowie das allgemeine Leben betreffende Konsequenzen wurden häufig als gravierendste Auswirkungen genannt und gleichzeitig im Schnitt auch besonders negativ bewertet. Die stärker negativen Bewertungen zu Beginn der Pandemie sowie zu Zeiten mit hohen Infektionszahlen und restriktiveren Pandemiemaßnahmen deuten darauf hin, dass eben genau während solcher kritischen Zeiten unterstützende Maßnahmen besonders relevant sind.

In den qualitativen Antworten wurde zudem ersichtlich, dass sich zentrale Themen im Verlauf der Pandemie verändern. Während zu Beginn der Pandemie beispielsweise besonders häufig der mangelnde Kontakt zum sozialen Umfeld aufgrund von Physical-Distancing-Anordnungen geäußert wurde, rückten im Verlauf eher eine anhaltende soziale Isolation, das Ende von Freundschaften durch die langen Abstandsphasen sowie soziale Spannungen durch unterschiedliche Ansichten zu SARS-CoV-2-Impfungen und anderen pandemierelevanten Themen in den Vordergrund. Auch medizinische Konsequenzen wurden – insbesondere durch die deutlich steigende Anzahl an bereits an COVID-19 erkrankten Menschen – zunehmend relevanter. Parallel dazu wurde beispielsweise auch der Wunsch nach medizinischer Unterstützung (z. B. in Bezug auf Long-COVID) häufiger.

Interessant ist des Weiteren, dass die gleichen Konsequenzen (z. B. Homeoffice) teils sehr negativ, teils aber auch ausgesprochen positiv empfunden wurden. Dies ist ein Indiz dafür, dass die subjektive Bewertung der Auswirkungen der Pandemie deutlich wichtiger ist als ihre objektive Einschätzung. Zudem ist es erwähnenswert, dass die Konsequenzen in der untersuchten Stichprobe im Durchschnitt weniger schlimm ausfielen als erwartet. Dies ist vereinbar mit der Beobachtung, dass gerade zu Beginn herausfordernder neuer Situationen Ängste und Befürchtungen – z. B. in Bezug auf die Konsequenzen – besonders ausgeprägt ausfallen und sich häufig im weiteren Verlauf nicht bewahrheiten und verringern (Bendau et al. 2021a). Dennoch sollte entsprechenden Sorgen im Vorhinein sowie tatsächlich erlebten negativen Konsequenzen die erforderliche Aufmerksamkeit geschenkt werden. Im Querschnitt waren negativere Konsequenzen mit höherer psychischer Belastung assoziiert, und auch im Längsschnitt einen Monat sowie ein Jahr später zeigten sich derartige Zusammenhänge zumindest für einzelne Variablen. Dies ist konsistent mit bisherigen Befunden (Henssler et al. 2021; Shevlin et al. 2020; Leung et al. 2022) und weist darauf hin, dass negative Auswirkungen der Pandemie auf verschiedene Bereiche des Lebens ernst genommen und, insofern möglich, präventiv und therapeutisch abgemildert werden sollten. Gleichzeitig gilt es aber zu beachten, dass die beobachteten Zusammenhänge eher klein ausfallen sowie ihre Bedeutsamkeit entsprechend vorsichtig interpretiert und in weiteren Studien überprüft werden sollten.

Unsere Daten weisen darauf hin, dass gerade psychotherapeutischer Unterstützung im Kontext der Pandemie ein hoher Stellenwert zuteilwird. Neben dem Bedarf von Einzeltherapien wurde häufig der Wunsch nach Gruppentherapien, ggf. auch online, geäußert. Des Weiteren wurden niedrigschwellige Angebote genannt, wie beispielsweise Online-Leitfäden, und Ratgeber zum Umgang mit pandemiebedingten Belastungen, Raum für gegenseitigen Austausch sowie eine größere Sichtbarmachung bestehender Angebote. In Bezug auf die Medien wurde zudem wiederholt der Appell nach etwas ausgewogeneren und weniger rein negativen Berichterstattungen geäußert. Diese Wünsche/Bedürfnisse zeigen auf, dass im weiteren Verlauf der COVID-19-Pandemie sowie möglicherweise zukünftigen (Gesundheits‑)Krisen Unterstützungsmaßnahmen in ganz verschiedenen Bereichen, Umfängen und Modalitäten hilfreich sein könnten. Natürlich ist die konkrete Implementation derartiger Angebote deutlich komplexer und von weiteren Faktoren abhängig, die erhobenen Daten geben aber zumindest einen Anhaltspunkt, dass ein substanzieller Anteil der Menschen im Pandemiekontext Unterstützung wünscht und dafür, welche Unterstützungsoptionen besonders gefragt sind.

Zudem scheinen nicht alle Unterstützungswünsche nur auf die Pandemie bezogen; einige bestanden möglicherweise bereits vor Beginn der Verbreitung von COVID-19. So wurden in Befragungen in der Allgemeinbevölkerung in Deutschland vor der Pandemie beispielsweise des Öfteren Wünsche nach vertiefteren und übersichtlich aufgearbeiteten Information zu verschiedenen Gesundheitsthemen (Hapke et al. 2010), sozialer Unterstützung und emotional-supportiver psychotherapeutischer Unterstützung (Braungardt et al. 2020) geäußert. Für einen direkten Vergleich unserer Studienergebnisse mit präpandemischen Daten liegen jedoch keine entsprechenden Untersuchungen vor.

### Reflexion und Ausblick

Unsere Studie zeichnet sich durch die längsschnittliche Mixed-methods-Analyse einer großen Stichprobe mit 10 Messzeitpunkten über 2 Jahre hinweg aus. Als eine Limitation gilt es, gleichzeitig jedoch zu berücksichtigen, dass durch das korrelativ-observationale Studiendesign sowie den Mangel an präpandemischen Daten keinerlei kausale Schlussfolgerungen möglich sind und Einflüsse von Drittvariablen auf die Ergebnisse nicht ausgeschlossen werden können. Des Weiteren wurden alle Variablen im Selbstbericht erfasst und können möglicherweise Erinnerungs‑, Wahrnehmungs- und Antwortverzerrungen unterliegen. Unsere Gelegenheitsstichprobe entspricht zudem in der Zusammensetzung nicht gänzlich der Allgemeinbevölkerung in Deutschland (höherer Anteil an weiblichen, jüngeren, hochgebildeten sowie geimpften Teilnehmenden), wodurch die Generalisierbarkeit unserer Ergebnisse limitiert wird. Zugunsten der Praktikabilität und Stichprobengröße wurden qualitative Aussagen schriftlich und umfangsreduziert erfasst; zukünftige Studien könnten noch ergänzend ausführliche qualitative Interviews führen und in quantitativen Analysen weitere (Dritt‑)Variablen integrieren. Für derartige zukünftige Studien und praktische Implikationen bietet die vorgestellte Studie eine gute Basis.

## Fazit für die Praxis

- Sozial-gesellschaftliche und das allgemeine Leben betreffende Konsequenzen der COVID-19-Pandemie werden im Schnitt besonders gravierend und negativ erlebt.
- Insbesondere Physical Distancing bzw. soziale Isolation, Veränderungen im Berufs- und (Hoch)Schulleben sowie negative Effekte auf die psychische Gesundheit wurden als besonders gravierende Auswirkungen der Pandemie genannt.
- Negativer erlebte Konsequenzen sind quer- und teilweise auch längsschnittlich mit stärkeren Angst- und depressiven Symptomen assoziiert.
- Unterstützungsmöglichkeiten im Kontext der Pandemie können vielfältig aussehen. Psychiatrisch-psychotherapeutische Unterstützung kann sich beispielsweise von der Bereitstellung kurzer Tipps, Leitfäden und Ratgeber bis hin zu Einzel- und Gruppentherapien erstrecken.
- Auch auf anderen Ebenen, wie beispielsweise einer ausgewogeneren Medienberichterstattung, ggf. mit psychoedukativen Elementen, ist Unterstützung erwünscht.
